# Coupling between prefrontal brain activity and respiratory sinus arrhythmia in infants and adults

**DOI:** 10.1016/j.dcn.2022.101184

**Published:** 2022-11-30

**Authors:** Trinh Nguyen, Stefanie Hoehl, Bennett I. Bertenthal, Drew H. Abney

**Affiliations:** a Department of Developmental and Educational Psychology, University of Vienna, Liebiggasse 5, 1010 Vienna, Austria; b Neuroscience of Perception and Action Lab, Italian Institute of Technology (IIT), Viale Regina Elena 291, 00161 Rome, Italy; c Department of Psychological and Brain Sciences, Indiana University Bloomington, 1101 E. 10th St., Bloomington, IN 47405, United States; d Department of Psychology, University of Georgia, 110 Hooper Street, Athens, GA 30602, United States

**Keywords:** Respiratory-sinus arrhythmia, fNIRS, Prefrontal cortex, Vagus nerve, Self-regulation, Brain-body connection

## Abstract

Self-regulation is an essential aspect of healthy child development. Even though infants depend on their caregivers for co-regulation during the first years, they begin to gain regulatory abilities through social interactions as well as their own developing agency and inhibitory control. These early regulatory abilities continue to increase with the development of both the prefrontal cortex and the vagal system. Importantly, theoretical accounts have suggested that the prefrontal cortex and the vagal system are linked through forward and backward feedback loops via the limbic system. Decreased coupling within this link is suggested to be associated with psychopathology. The primary goal of this study was to examine whether intrapersonal coupling of prefrontal brain activity and respiratory sinus arrhythmia is evident in infancy. Using the simultaneous assessment of functional near-infrared spectroscopy and electrocardiography, we used Cross-Recurrence Quantification Analysis to assess the coupling of prefrontal brain activity and respiratory sinus arrhythmia in 69 4- to 6-month-old infants and their mothers during a passive viewing condition. However, we did not find significant coupling between the PFC and RSA in infants and adult caregivers. Future studies could examine social contexts associated with greater emotional reactivity to deepen our understanding of the pathways involved in self-regulation.

Self-regulation is a critical aspect of children’s socio-cognitive development. During the first years, infants depend on their caregivers to co-regulate when an emotional challenge occurs (Porter, 2003). Still, infants show early regulatory abilities, as these abilities allow them to cope with distressing instances of parents’ unresponsiveness in their daily social interactions (Markova and Nguyen, 2022). Theoretical accounts have identified the prefrontal control of limbic regions (including the cingulate, insular cortices, and the amygdala) as centrally involved in self-regulation (Ochsner and Gross, 2007, Perlman and Pelphrey, 2010). Moreover, the prefrontal cortex (PFC) is involved in a pathway connected to the vagus nerve via feedforward and feedback connections (Beauchaine, 2015). One common index of vagal activity is respiratory sinus arrhythmia (RSA). Notably, several forms of psychopathology are characterized by different types of PFC dysfunction, including both heightened PFC and reduced PFC responses (Maren et al., 2013, Menon, 2011). In addition, low basal RSA and excessive or diminished RSA reactivity are peripheral biomarkers of poor executive control (Black et al., 2021, Thayer et al., 2009). Still, we know surprisingly little about the connection between the PFC and striated vagus nerve in infants as well as adults and the role it plays in healthy neurophysiological development. In the current study, we investigated the functional relation between the PFC and the vagal system in infants and their mothers during a passive viewing task.

Human infants are not born with a fully developed myelinated vagus. Instead, vagal development continues in the first few months postpartum (Porges and Furman, 2011). Myelinated vagal fibers significantly increase between 30‐32 weeks gestational age and six months postpartum (Sachis et al., 1982). During this time, vagal tone steadily increases and then stabilizes as the vagal fibers continue to increase (Izard et al., 1991, Porges and Furman, 2011, Porter et al., 1995). As the myelinated vagal fibers increase with development, visceral regulation improves, and this has been observed to be associated with infants starting to display enhanced behavioral and emotion regulation (Porges and Furman, 2011). Empirical evidence also reveals that infants’ regulatory abilities are characterized by individual differences during the first half year. At four months of age, only around 50% of infants show vagal suppression (RSA decrease) upon emotional challenge (Abney, daSilva, and Bertenthal, 2021). Therefore, the first half year after birth represents a key period for the development of self-regulation through increasing vagal activity.

It is well established that older children and adults with attenuated self-regulation capacities (excessive or dampened emotional reactivity or heightened distress/anxiety) often display low RSA tone during resting phases and excessive or blunted RSA reactivity to emotional challenge (Beauchaine, 2015, Black et al., 2021). Those individuals may display various forms of internalizing and externalizing psychopathology, and specific psychopathological syndromes, including anxiety, phobias, and depression (Beauchaine and Thayer, 2015). Emerging evidence suggests that low RSA and excessive/blunted RSA reactivity index poor self-regulation because they are downstream peripheral markers of PFC dysfunction. The medial prefrontal cortex (mPFC) is connected to the parasympathetic nervous system via bidirectional pathways (see Fig. 1 for a schematic outline; Beauchaine, 2015). The prefrontal, cingulate, insular cortices, the hypothalamus, and the amygdala form a neural network (Thayer et al., 2009). The amygdala within this network is connected to the locus coeruleus, which in turn is connected to the nucleus solitary tract. These structures provide input via the parasympathetic nervous system to the sinoatrial node, which is the primary pacemaker of the heart (Porges, 1995). Taken together, cognitive appraisal modulates the pathways between the mPFC and the parasympathetic nervous system, which can result in reduced basal RSA and excessive or blunted RSA reactivity in case of poor modulation (Beauchaine, 2015). Importantly, these structural pathways between the mPFC and the parasympathetic nervous system are based on findings in adults, while it remains unclear if these same pathways are functional in infants. Still, even in adults, empirical evidence for the connection between the mPFC and RSA is sparse. However, preliminary evidence shows that heart rate variability is associated with amygdala-mPFC functional connectivity and mPFC activity during rest, underscoring neurovisceral involvement in self-regulation (Lane et al., 2009, Sakaki et al., 2016). However, to our knowledge, no prior work has examined the concurrent relation between the mPFC and RSA in children or infants. Accordingly, we assume that as the mPFC and its functional connections develop throughout the first year of life, an association with the vagal system should increase. Brain development, together with social experiences, could thus be associated with self-regulation, mediated by PFC-RSA coupling.

**Fig. 1:**
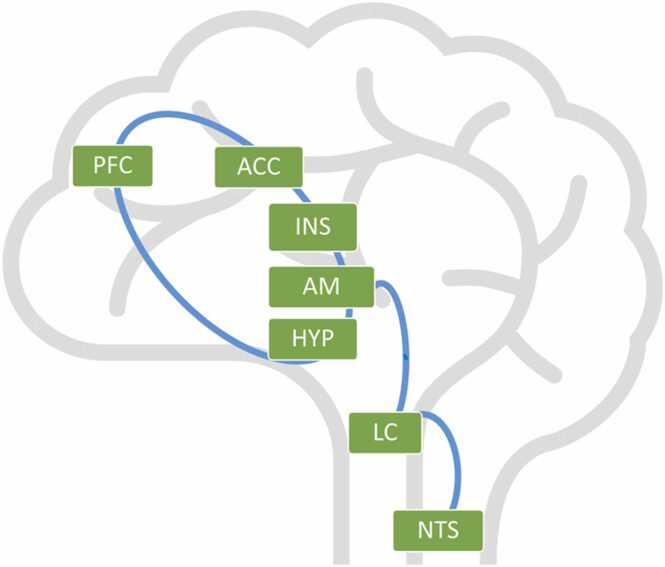
Schematic outline of the bidirectional connection between the prefrontal cortex (PFC), the anterior cingulate cortex (ACC), the insula (INS), the amygdala (AM), and the hypothalamus (HYP). The network is connected to the locus coeruleus (LC), which is connected to the nucleus solitary tract (NTS).

The PFC is related to numerous social and non-social cognitive functions and plays a fundamental role in understanding self and others (Frith and Frith, 2006). Previously, the PFC was assumed to be functionally silent during the first year of life. However, a growing body of research shows that functional PFC development and involvement are visible from birth (e.g., Grossmann, 2013). Evidence suggests that the PFC is involved in multiple cognitive processes, such as speech and language, executive control, and mentalizing. These processes are essential for social cognition and interaction. While the role of PFC in self-regulation at preschool age is well researched (Grabell et al., 2019, Perlman et al., 2014, Perlman and Pelphrey, 2010), there is less evidence regarding the involvement of the PFC in self-regulation during infancy. Still, for instance, cerebral blood flow in 5- to 8-month-old infants in frontal regions was positively related to temperamental negative affectivity and recovery of positive affect after a stressor (Catalina Camacho et al., 2020). These preliminary findings highlight the relevance of the PFC for self-regulation at both trait (i.e., temperament) and state levels.

The first year of life plays an essential role in PFC development, as structural and functional alterations of the PFC become visible during this time. Premature birth, illness, or neglect may attenuate a healthy (neuro-) developmental trajectory (Porges and Furman, 2011). Atypical development could put infants at risk for lower sensitivity to social cues (see Grossmann, 2013), which is also modulated by the vagal system. The question then remains whether atypical development of either the PFC, the vagal system, or both might inhibit the growing connection between the PFC and striated vagal nerve and consequently healthy child development. Infant temperament offers a look into early individual differences in affective and behavioral traits (Rothbart et al., 2011). More specifically, infants’ temperamental traits are defined in terms of reactivity and regulation and are often assessed using parental reports as well as laboratory measures (Sidor et al., 2017, Tang et al., 2020). Accordingly, there is growing evidence suggesting a connection between temperament and RSA as well as PFC activity (Fox, 1989, Kelsey et al., 2021, Kelsey et al., 2021, Morales et al., 2015). For instance, connectivity in the aforementioned prefrontal, cingulate and insular network with the amygdala in newborns was associated with fear and cognitive development at 6 months (Graham et al., 2016, Thomas et al., 2019). Connectivity within this network has been specifically linked to infant temperament profiles, suggesting that a balance between temperamental negative affectivity and cognitive skills in infants allows them to efficiently self-regulate (Degnan and Fox, 2007, Nigg, 2006). We, therefore, propose that variance in infants’ coupling between PFC and RSA is related to infants’ temperament. Understanding the PFC-vagal system connection is of utmost importance to help identify and support infants at risk for developing difficulties in behavioral state regulation, poor affective tone, and diminished abilities for reciprocal social engagement behaviors.

## 1 Current study

The primary goal of this study is to examine covariation in PFC activity and vagal reactivity in infants at 4 to 6 months of age compared to an adult sample, comprised of infants’ mothers. We concurrently assessed PFC activity and vagal tone as assessed via RSA during a resting phase of a multi-level paradigm in both infants and caregivers. Brain activity was continuously assessed using functional near-infrared spectroscopy (fNIRS) in medial and lateral PFC as well as bilateral inferior frontal gyrus. RSA was continuously assessed using electrocardiography and a novel dynamic sliding-window processing method. Our goal was to assess the association between brain activity and vagal tone in both infants and their adult caregivers over the course of a resting phase (i.e., passive viewing condition) to test for non-random coupling between PFC and RSA activity. To do so, we conducted the following set of tests:

1) We tested the covariation between PFC and RSA activity, predicting that increases and decreases in PFC activity would be related to increases and decreases in RSA activity during a resting phase.
2) We tested whether and how coupling between PFC and RSA activity maps onto individual differences in infants’ temperament (negative affectivity, surgency, effortful control) and infants’ positive and negative affect during a free play situation with their mother. We hypothesized that higher PFC-RSA coupling is related to lower parent-reported negative affectivity and higher surgency and effortful control. We also predicted that higher PFC-RSA coupling is related to higher durations of positive affect and lower durations of negative affect during play.
3) We tested whether this PFC-Vagal system connection is related to individuals’ basal RSA. Here, we suggested that low basal RSA will be associated with decreased covariation between PFC activity and RSA reactivity, as the individuals’ RSA reactivity is restricted by the lower physiological boundaries of the vagal system. In infants, we further tested whether there is an age-related increase in the strength of the PFC-RSA connection.

## 2 Methods

### 2.1 Sample characteristics

Overall, 69 mother-infant dyads completed the experiment and were recruited from a database of volunteers. Infants’ age ranged from 4- to 6-months-old (*M* = 4.8 months; *SD* = 16 days; 33 girls). Infants were born healthy and at term, with a gestation period of at least 36 weeks. Mothers’ age averaged 33.97 years (*SD* = 4.94) and 70.1% of mothers had a university degree. All dyads were of White European origin and came from middle to upper-class families based on parental education. All infants and mothers had no neurological problems as assessed by maternal report. An additional 11 infants participated in the present study but were excluded due to bad signal quality, fussiness (infants started to cry during the preparation phase or before the end of the experiment), and sleepiness. Included participants did not differ from the excluded participants in terms of infants’ age, biological sex or maternal age and education, *p* > .051.

### 2.2 Experimental procedure

**Fig. 2:**
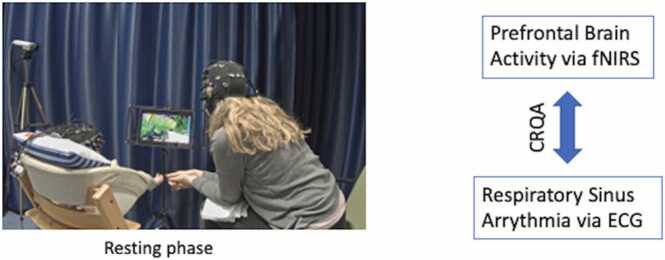
Exemplary dyad in the resting phase. Prefrontal brain activity and respiratory sinus arrhythmia were concurrently measured in both infant and adult caregiver and subsequently assessed with regard to whether the two time-series co-varied (using Cross-Recurrence Quantification Analysis [CRQA]) during rest (90 s).

During the experiment, caregiver and infant were either seated next to one another (resting phase; Fig. 2) or the infant sat on the caregiver's lap as both were watching a calm aquarium video on a tablet (proximate watching conditions). The videos lasted 90 s and depicted fish swimming in a tank. The order of the two viewing conditions was counterbalanced. Next, mother and infant engaged in a 5 min long free play without toys and song while both were seated face-to-face. For the purposes of this study, we consider the following experimental conditions: (1) the resting phase and (2) the free play condition to assess infants’ behaviors. Neural activity in the mother-infant dyad was simultaneously measured with functional near-infrared spectroscopy. We assessed respiratory sinus arrhythmia (RSA) with signals recorded by electrocardiography (ECG), and each dyad was filmed by three cameras (angled towards the dyad, the infant, and the mother) throughout the experiment. Recent research with the same paradigm and condition durations (90 s) showed robust coupling of neural activity and RSA between infants and caregivers using similar measures and statistical techniques (Nguyen et al., 2021).

### 2.3 Data acquisition

#### 2.3.1 fNIRS Recordings

We used two NIRSport 8–8 (NIRx Medizintechnik GmbH, Germany) devices to simultaneously record oxy-haemoglobin (HbO) and deoxy-haemoglobin (HbR) concentration changes in mother and infant. The 8 × 2 probe sets were attached to an EEG cap with a 10–20 configuration (Fig. S1). The standard electrode locations allowed us to place the probes more precisely so that they were located over the left and right inferior frontal gyrus (IFG) corresponding to F7 and F8, whereas the probes on the medial prefrontal area (mPFC) corresponded to FP1 and FP2 *(*Fig. S2*)*. These regions of interest were based on previous work involving adult-child interactions (Nguyen et al., 2020, Piazza et al., 2020, Redcay and Schilbach, 2019). In each probe set, 8 sources and 8 detectors were positioned, which resulted in 16 measurement channels with equal distances of ∼2.3 cm between the infants’ optodes and 3 cm between the mothers’ optodes. The absorption of near-infrared light was measured at the wavelengths of 760 and 850 mm, and the sampling frequency was 7.81 Hz.

### 2.4 Electrocardiogram (ECG) Recordings

We used a Brain-Amp system (Brain Products GmbH, Germany) with two amplifiers to measure two standard single-channel ECG registrations (lead II derivation). One electrode was placed on the upper right chest, one on the left side of the abdomen, and the grounding electrode was placed on the right side of the abdomen on both infant and mother. The ECG signal was recorded with a 500 Hz sampling frequency. Interbeat-intervals (IBIs) were then extracted offline using ARTiiFACT (Kaufmann et al., 2011). The ECG data were visually inspected for correct detections and artifacts by trained research assistants. When ectopic beats or erroneous detections were found, the data were semi-automatically corrected (removal of erroneous detection/artifact followed by a cubic spline interpolation; corrections < 1%).

### 2.5 Parent-reported infant temperament

Infant temperament was assessed by parent-report utilizing the very short form of the revised Infant Behavior Questionnaire (IBQ; Putnam et al., 2014). The questionnaire assesses three infant temperament subscales in 3- to 12-month-old infants. The subscales are surgency, effortful control, and negative affectivity. The subscale surgency consists of items concerning the infants’ approach, vocal reactivity, high-intensity pleasure, smiling and laughter, activity level, and perceptual sensitivity. The subscale negative affectivity consists of items regarding sadness, distress to limitations, fear, and rate of recovery from distress. The subscale orienting/regulation consists of items regarding low-intensity pleasure, cuddliness, duration of orienting, and soothability (Gartstein and Rothbart, 2003). Mothers rated the frequency that their infant engaged in specific day-to-day behaviors in the prior one to two weeks using a 7-point scale, with responses ranging from 1 (never) to 7 (always).

### 2.6 Behavioral coding of infants’ affect

To assess infants’ affect, a potential marker for instances of self- and co-regulation during caregiver-child interactions, trained graduate students coded video recordings of the free-play sessions using Mangold INTERACT. The experimental sessions were filmed at 25 frames per second. Infants’ facial affect was micro-coded frame-by-frame for duration and frequency. We distinguished between positive facial expressions (smiling with mouth turned upward (open or closed), contraction of cheek muscle and/or under-eye muscle), negative facial expressions (distress, fretting, anger, discontent, sadness or “pout face” as indexed by narrowed eyes, mouth curled or grimacing, lowered brows, mouth corners turned down) and neutral facial expressions. We calculated inter-rater reliability in 25% of randomly chosen videos using kappa, which resulted in overall k = 0.81 for facial affect. To control for minor variations in interaction duration (*M*=291.36 s, *SD*=38.08 s), the total duration of each facial affect category was divided by the total time of free-play (i.e., duration of coded free-play) to be able to control for different interactional durations between dyads. The affect scores thus indicate proportions of time during each condition.

#### 2.6.1 Analysis pipeline

fNIRS measurements were processed using MATLAB-based functions derived from Homer 2 (Huppert et al., 2009). Raw data were automatically pruned using the enprunechannels function (dRange = [0.03 2.5]; SNRThresh = 10) and subsequently converted into optical density. Next, optical density data were motion-corrected with a wavelet-based algorithm with an interquartile range of 1.5 (Molavi and Dumont, 2012). Motion-corrected time series were further visually inspected during a quality check procedure. We checked frequency-time plots of time-series for integrity, a clearly visible heart band, and visually observable motion artifacts (Nguyen, Hoehl et al., 2021) resulting in the removal of 22.87% of channels of infants and caregivers. Then slow drifts and physiological noise were removed from the signals using a band-pass second-order Butterworth filter with cutoffs of 0.01 and 0.5 Hz and a slope of 12 dB per octave. The filtered data were converted to HbO and HbR values (μMol) based on the modified Beer-Lambert Law. For the proposed secondary data analysis, HbO and HbR time-series were down-sampled to 5 Hz to match the sampling rate of RSA.

IBIs were down-sampled to 5 Hz, and a 51-point band-pass local cubic filter was used to estimate and remove the slow periodic and aperiodic components of the time series (Abney, daSilva, Lewis et al., 2021). A FIRtype bandpass filter was applied to further isolate the variance in the IBI series to only the frequency range of spontaneous breathing for infants (0.3–1.3 Hz) and adults (0.12–1.0 Hz). The higher range of 1.0 Hz for mothers’ respiration was used to account for the infrequent occurrence of faster breathing during talking or playing segments so that the same filter could be used for all mothers in all conditions. The Porges & Bohrer (Porges and Bohrer, 1990) technique for RSA magnitude estimation includes parsing this component signal into discrete epochs (lasting 10–120 s), then calculating the natural log of the variance in each epoch. RSA is reported in units of ln(ms)². Instead of following the Porges & Bohrer method, we introduced a sliding window of 15 s that was updated every 200 ms in order to extract a more continuous estimate of cardiac vagal tone for both participants. The estimated RSA value corresponds to the first value of the sliding window. RSA values were then detrended by calculating the change values of RSA over the course of the resting phase (see Abney et al., 2021 for more details).

To be clear, the fNIRS and RSA time series were either down-sampled or sampled at 5 Hz. It remains an open question as to the optimal timescale for determining covariations of signals that are likely fluctuating at different (or multiple) timescales. We chose the free parameter of sample rate of 5 Hz because this is a timescale (milliseconds) that is fast enough to capture known response dynamics of hemodynamic fluctuations of fNIRS and respiratory fluctuations of RSA. Moreover, the analytic procedure that we discuss next allows for the detection of maximum covariation within a window size of 10 s, which provides a conservatively large time window for observing lagged covariations.

We used Cross-Recurrence Quantification Analysis (CRQA) to calculate the coupling between prefrontal brain activity (HbO and HbR) in three different brain areas (IFG vs. lPFC vs. mPFC) and RSA change values (Coco and Dale, 2014, Konvalinka et al., 2011). Coupling was calculated during the resting phase, which lasted 90 s. CRQA is a nonlinear method for analyzing shared dynamics between two different data series (Shockley et al., 2002) and has been applied successfully to investigate cardio-respiratory dynamics (Konvalinka et al., 2011, Nguyen et al., 2021) and behavioral coordination (Abney et al., 2015). The method is especially suitable for neurophysiological coupling estimations, as it does not assume stationarity within the data. Broadly, CRQA provides information about how often two signals co-visit areas in a state-space that is inferred from the measured time series. When two signals co-visit the same areas of a state-space at approximately the same time, this increased temporal coordination is reflected in high recurrence rates. We can also investigate long-range patterns of influence across two signals using CRQA by quantifying the similarity of signal patterns that are separated in time.

We used one metric to evaluate neurophysiological coupling: the maximum recurrence (MAX REC) from the cross-recurrence profile. MAX REC is a metric that assesses the proportion of time two signals are in a similar region of a state space and is generally considered as an overall estimate of coordination: Higher MAX REC estimates are interpreted as two signals having higher degrees of coordination. MAX REC is an ideal metric for the purposes of assessing coordination of two signals (neural activity and RSA) that are likely fluctuating at different timescales. MAX REC provides information about the coordination patterns between neural and physiological signals and also addresses potential differences in the response dynamics and timescales of the two different signals. We utilized the *drpdfromts* function from the **crqa** package (Coco and Dale, 2014) and set the window size to 10 s, radius to .02, embedding dimension(s) to 1, and the delay to 16 to be consistent with our previous research using CRQA on similar signals (Nguyen, Abney et al., 2021). The radius parameter determines how far into a state space two points can be from each other to still be considered recurrent. The delay parameter sets how many points in time to consider when determining if points are recurrent. The embedding dimension parameter sets how many states must match to be counted as recurrent. For the statistical analyses, our dependent measure was MAX REC. Statistical analyses involving neurophysiological coupling focused on HbO and RSA values, while coupling analyses between HbR values and RSA are included in the supplemental materials.

Control analysis. We compared REC between observed PFC-RSA coupling and random pairings of PFC and RSA time series of participants to ensure that our measure of neurophysiological coupling was not a function of chance. By establishing a baseline of coupling through surrogate analysis (e.g., Abney et al., 2015; Nguyen, Abney et al., 2021; Nguyen, Hoehl et al., 2021), we can compare properties of the observed PFC-RSA coupling against what might be expected by chance / spurious correlation. For the surrogate test, we created surrogate data series combinations by randomly pairing the neural time series of each participant with the RSA time series of other participants. This provided a baseline for the rates of recurrence that could be expected by chance or the amount of similarity of behavior that we might expect as a function of the environment or task rather than the actual process of coupling. We created 400 surrogate pairs (each PFC time series was paired with 400 randomly selected RSA time series from the same sample) and then performed CRQA on each pairing to estimate recurrence profiles. The control analysis was conducted for both the infant and adult samples, respectively.

To statistically compare the rates of recurrence between observed PFC-RSA coupling and random surrogate coupling, we performed generalized linear mixed effects modeling. Two separate models were estimated: one for infants (Infant Model 1) and one for adults (Adult Model 1). MAX REC was added as the response variable and modeled over fixed effects of pairing (true vs. random), region (IFG vs. lPFC vs. mPFC), and the interaction between pairing and region. From these models, we could conclude whether the observed levels of PFC-RSA covariation, as viewed through CRQA, are significantly above spurious covariation.

The following covariates were considered. First infants’ temperament (i.e., negative affectivity, surgency, effortful control), assessed by parent-report and infants’ positive and negative affect during the free play condition were tested as covariates in PFC-RSA coupling. Second, basal RSA was estimated by calculating the average RSA over the resting phase. Next, infants age in days was included. To test the covariates, we ran three additional models. Model 2 tested the effects of infants’ temperament and behavior on their intrapersonal coupling of PFC and RSA activity. MAX REC was added as the response variable and modelled over fixed effects of region, infant negative affectivity, surgency, effortful control, proportion of durations of infants’ positive and negative affect. Infant Model 3 tested the effects of infants’ basal RSA and age on PFC-RSA coupling. MAX REC was added as the response variable and modelled over fixed effects of region, infants’ basal RSA and age. As Model 1 did not display regional differences in intrapersonal PFC-RSA coupling, we did not include interaction effects between region and the covariates, respectively. Adult Model 3 tested the effects of the adults’ basal RSA on their intrapersonal coupling of PFC and RSA activity. MAX REC was added as the response variable and modelled over fixed effects of region and basal RSA. Again, as Model 1 did not display regional differences in intrapersonal PFC-RSA coupling, we did not include interaction effects between region and basal RSA.

We attempted to include a full random effects structure in all models, but the model estimations did not converge. The random effects structure of each model thus only included a random intercept for the individuals.

### 2.7 Previous author involvement

Author T.N. was involved in data collection, preprocessing, and previous analyses (analyses on interpersonal neural and physiological synchrony as well as maternal touch), will thus provide author D.H.A. with fNIRS and ECG pre-processed data to ensure unbiased data analyses. All following data and statistical analyses were conducted by D.H.A., who was not involved in data collection and previous analyses. All authors were involved in writing a manuscript concerning the role of proximity and touch in neural and physiological synchrony in naturalistic mother-infant interaction, drawing from the same dataset as reported here.

Nguyen, T., Abney, D. H., Salamander, D., Bertenthal, B. I., & Hoehl, S. (2021). Proximity and touch are associated with neural but not physiological synchrony in naturalistic mother-infant interactions. *NeuroImage*, *244*, 118599. https://doi.org/10.1016/j.neuroimage.2021.118599.

### 2.8 Statistical Power Analysis

We conducted a statistical power analysis using a bespoke web application (Judd et al., 2017). We entered a sample size of 65, considering further dropouts due to low data quality. Previous research with neural correlates of heart rate variability displayed large effect sizes (d > 0.8) (Lane et al., 2009, Sakaki et al., 2016). Our main research question was to test above-threshold coupling between changes in PFC and RSA. We, therefore, conducted the power analysis assuming large and moderate effect sizes (d=0.5–0.8), which resulted in 1-β = 0.871–0.999. Accordingly, our sample size is appropriate for the planned analysis.

## 3 Stage 2 Report

### 3.1 Results

#### 3.1.1 Confirmatory analyses

##### 3.1.1.1 Control analyses

We first tested the covariation between PFC and RSA activity, predicting that increases and decreases in PFC activity would be related to increases and decreases in RSA activity during a resting phase. We, therefore, compared the rates of recurrence between observed coupling to random surrogate coupling in infants (Infant Model 1) and adult caregivers (Adult Model 1). The descriptive statistics and full model outputs are included in the Supplements (Table S1, S2). The results showed no significant differences between IFG vs. lPFC vs. mPFC (frontal-cortical)-RSA coupling in true pairings and random surrogates in infants, *p* > .206. Results from the adult model also showed no significant differences between coupling in true pairings and random surrogates, *p* > .110. We, therefore, found neither significant PFC-RSA coupling in infants nor in adults. As Infant and Adult Model 1 did not reveal significant differences between brain regions of interest, we excluded an interaction effect between regions and other covariates in further analyses.

##### 3.1.1.2 Covariate analyses

Next, we ran Models 2–3 to test whether PFC-RSA coupling in infants and adults was related to infant and adult related covariates. Model 2 tested the effects of infant temperament and behavior on infants’ intrapersonal coupling of frontal-cortical and RSA activity. None of the infant temperament subscales (negative affectivity, surgency or effortful control) nor behaviors (positive and negative affect) were significantly related to infant neurophysiological coupling, *p* > .199. Infant Model 3 tested the effects of basal RSA, and infant age on neurophysiological coupling. Neither basal RSA nor infant age were significantly related to infant neurophysiological coupling, *p* > .052. Adult Model 3 tested the effects of the adult basal RSA on adults’ intrapersonal coupling of frontal-cortical and RSA activity. Again, we did not find any significant effect of adult basal RSA, *p* > .172.

##### 3.1.1.3 Exploratory analyses

In exploratory analyses we tested the same models on PFC-RSA coupling during a free play interaction instead of the baseline condition, as we assumed there would be more variance in both PFC and RSA activity, potentially facilitating coupling processes. Infant Model FP1 thus compared recurrence rates between observed neurophysiological coupling to random surrogate coupling in infants. The model output showed that recurrence rates were not significantly different from one another, *p* > .266. Adult Model FP1 tested the same comparison in adult caregivers. Again, the model revealed no significant differences between observed and random coupling in adult caregivers, *p* > .142. We, therefore, did not conduct further analyses with covariates.

## 4 Discussion

Our goal was to assess the coupling between brain activity in inferior and prefrontal areas of the cortex and vagal tone in both infants and their adult caregivers over the course of a resting phase to further understand the pathways involved in self-regulation. We were, however, unable to identify significant PFC-RSA coupling in either infants or adults; moreover, any association with individual variables, such as infant temperament, age or both infant and adult basal RSA were not significant. In addition, we did not find coupling between cortical and vagal processes during a face-to-face free play interaction. We now discuss possible reasons for not observing PFC-RSA coupling in either infants or adults.

Conceivably, these interaction contexts might not have induced enough variation nor the need for PFC-RSA coupling (as indicated in the descriptive statistics, Table S1). Coupling is more likely to occur during periods of heightened arousal and/or need for regulation, as a function of stress (Beauchaine, 2015, Black et al., 2021). Our paradigm was not designed to stress or excite infants and might therefore not have induced sufficient coupling to be detected in this study. A more suitable paradigm should be employed to directly measure self-regulation during and following an exciting or stressful experience, such as game routines (Markova, 2018), delay of gratification (Mischel et al., 1989) or the Still Face paradigm (Tronick et al., 1978),.

Let’s consider what happens during the Still-Face Paradigm which simulates the interruption of coordinated infant–parent interaction. After a period of face-to-face dyadic interaction, the parent is instructed to cease responding to the infant with an inexpressive, unresponsive face known as the “still face.” After this disruption social interaction resumes in the “reunion” phase. Infants tend to significantly increase negative affect from face-to-face play to the still face, and then decrease negative affect once maternal interaction resumes in reunion (Mesman et al., 2009). These behavioural changes are accompanied by changes in physiological state across phases: infants typically increase heart rate and decrease cardiac vagal tone in response to the still face (suggesting a decrease in safety-related parasympathetic regulation), followed by subsequent decreases in heart rate and increases in vagal tone during reunion (Abney et al., 2021, Jones-Mason et al., 2018). These physiological changes are mediated by the myelinated vagal pathways providing a parasympathetic influence on the heart that increases or decreases depending on whether mothers’ and infants’ behaviors are mutual and reciprocal or mismatched (Porges, 1995). As such, there are patterns of RSA change and recovery throughout the still face paradigm. By contrast, the experimental paradigm used in our study did not include any external challenges to infants. Consequently, RSA demonstrated much less variability (see Table S1, Abney, daSilva, and Bertenthal, 2021), making it difficult to observe any coupling with PFC activity.

While the selection of a non-stressful paradigm might explain why infants in our sample did not show PFC-RSA coupling, previous adult studies showed covariation along the aforementioned structural pathways between the PFC and the parasympathetic nervous system during rest (Lane et al., 2009, Sakaki et al., 2016). In particular, heart-rate variability was related to PFC activity and PFC-amygdala connectivity. As such, these findings suggest a further reason why we did not identify direct functional PFC-RSA coupling in adults or infants. Due to our fNIRS channel setup and the measurement device, per se, we were unable to measure additional brain regions, especially subcortical ones, such as the amygdala or the insula, which are suggested to be intermediary connections in this network (Sakaki et al., 2016). Future studies might consider functional magnetic resonance imaging (to assess subcortical regions) or pupillometry (as an indirect measure of locus coeruleus activity, Joshi et al., 2016) in order to monitor the activity of some of these intermediary regions.

In addition, methodological challenges could have also impacted our results. CRQA is a non-parametric analysis and might not be as sensitive as parametric analyses (Sheskin, 2011). Small effects might have gone undetected due to our sample size and limited recording period. Therefore, future studies should consider collecting data for longer periods of time and implementing other approaches to measuring coupling between time series on different scales (such as cross-frequency coherence, González et al., 2020).

Despite the limitations in our design, the data from this study suggest that there is no strong functional relation between hemodynamic PFC and RSA activity during safe and secure situations in infants and adults. While recent research has pointed towards heart-brain (Al et al., 2020) and gut-brain interactions (Kelsey, Prescott et al., 2021), neurophysiological processes might still work independently in certain cases. The interpersonal alignment of neural and physiological activity in interacting mother-child (Reindl et al., 2022) and mother-infant dyads (Nguyen, Abney et al., 2021) emerges during different contexts (cooperation for neural synchrony vs. competition for physiological synchrony) as well as in relation to different behaviors (touch for neural synchrony vs. facial affect for physiological synchrony), hinting at potentially different functions to the neural and physiological systems of interacting agents. Especially healthy, neurotypical participants, such as our sample, might show only sub-threshold coupling of PFC and RSA activity. Further research is needed to identify precisely when and why different functionalities of the PFC and RSA might be beneficial.

In conclusion, we did not find intrapersonal coupling between inferior and prefrontal brain activity and RSA, an indicator of vagal activity. The limitations in our study, however, constrain the sorts of generalizations that are possible. Future studies need to include situations associated with greater emotional reactivity, such as the still face, to deepen our understanding of the pathways involved in self-regulation.

## Data Statement

The Registered Report Protocol is available on OSF: 10.17605/OSF.IO/TPHAC.

## Declaration of Competing Interest

The authors declare that they have no known competing financial interests or personal relationships that could have appeared to influence the work reported in this paper.

## Data Availability

Preprocessed fNIRS and RSA data, data processing and analysis scripts are made available on OSF: https://osf.io/sr6hf/.

## References

[bib1] Abney D.H., Paxton A., Dale R., Kello C.T. (2015). Movement dynamics reflect a functional role for weak coupling and role structure in dyadic problem solving. Cogn. Process..

[bib2] Abney D.H., daSilva E.B., Lewis G.F., Bertenthal B.I. (2021). A method for measuring dynamic respiratory sinus arrhythmia (RSA) in infants and mothers. Infant Behav. Dev..

[bib3] Abney D.H., daSilva E.B., Bertenthal B.I. (2021). Associations between infant–mother physiological synchrony and 4- and 6-month-old infants’ emotion regulation. Dev. Psychobiol..

[bib4] Al E., Iliopoulos F., Forschack N., Nierhaus T., Grund M., Motyka P., Gaebler M., Nikulin V.V., Villringer A. (2020). Heart–brain interactions shape somatosensory perception and evoked potentials. Proc. Natl. Acad. Sci..

[bib5] Beauchaine T.P. (2015). Respiratory sinus arrhythmia: a transdiagnostic biomarker of emotion dysregulation and psychopathology. Curr. Opin. Psychol..

[bib6] Beauchaine T.P., Thayer J.F. (2015). Heart rate variability as a transdiagnostic biomarker of psychopathology. *Int. J. Psychophysiol.*, *98*(2, Part.

[bib7] Black C.J., Hogan A.L., Smith K.D., Roberts J.E. (2021). Early behavioral and physiological markers of social anxiety in infants with fragile X syndrome. J. Neurodev. Disord..

[bib8] Catalina Camacho M., King L.S., Ojha A., Garcia C.M., Sisk L.M., Cichocki A.C., Humphreys K.L., Gotlib I.H. (2020). Cerebral blood flow in 5- to 8-month-olds: regional tissue maturity is associated with infant affect. Dev. Sci..

[bib9] Coco M.I., Dale R. (2014). Cross-recurrence quantification analysis of categorical and continuous time series: an R package. Front. Psychol..

[bib10] Degnan K., Fox N. (2007). Behavioral inhibition and anxiety disorders: multiple levels of a resilience process. Dev. Psychopathol..

[bib11] Fox N.A. (1989). Perspectives on behavioral inhibition.

[bib12] Gartstein M.A., Rothbart M.K. (2003). Studying infant temperament via the Revised Infant Behavior Questionnaire. Infant Behav. Dev..

[bib13] González J., Cavelli M., Mondino A., Rubido N., BL Tort A., Torterolo P. (2020). Communication through coherence by means of cross-frequency coupling. Neuroscience.

[bib14] Grabell A.S., Huppert T.J., Fishburn F.A., Li Y., Hlutkowsky C.O., Jones H.M., Wakschlag L.S., Perlman S.B. (2019). Neural correlates of early deliberate emotion regulation: young children’s responses to interpersonal scaffolding. Dev. Cogn. Neurosci..

[bib15] Graham A.M., Buss C., Rasmussen J.M., Rudolph M.D., Demeter D.V., Gilmore J.H., Styner M., Entringer S., Wadhwa P.D., Fair D.A. (2016). Implications of newborn amygdala connectivity for fear and cognitive development at 6-months-of-age. Dev. Cogn. Neurosci..

[bib16] Grossmann T. (2013). Mapping prefrontal cortex functions in human infancy. Infancy.

[bib17] Huppert T.J., Diamond S.G., Franceschini M.A., Boas D.A. (2009). HomER: a review of time-series analysis methods for near-infrared spectroscopy of the brain. Appl. Opt..

[bib18] Izard C., Porges S., Simons R., Haynes O., Hyde C., Parisi M., Cohen B. (1991). Infant cardiac activity: developmental changes and relations with attachment. Dev. Psychol..

[bib19] Jones-Mason K., Alkon A., Coccia M., Bush N.R. (2018). Autonomic nervous system functioning assessed during the still-face paradigm: a meta-analysis and systematic review of methods, approach and findings. Dev. Rev..

[bib20] Joshi S., Li Y., Kalwani R.M., Gold J.I. (2016). Relationships between pupil diameter and neuronal activity in the locus coeruleus, colliculi, and cingulate cortex. Neuron.

[bib21] Judd C.M., Westfall J., Kenny D.A. (2017). Experiments with more than one random factor: designs, analytic models, and statistical power. Annu. Rev. Psychol..

[bib22] Kaufmann T., Sütterlin S., Schulz S.M., Vögele C. (2011). ARTiiFACT: a tool for heart rate artifact processing and heart rate variability analysis. Behav. Res. Methods.

[bib23] Kelsey C.M., Farris K., Grossmann T. (2021). Variability in infants’ functional brain network connectivity is associated with differences in affect and behavior. Front. Psychiatry.

[bib24] Kelsey C.M., Prescott S., McCulloch J.A., Trinchieri G., Valladares T.L., Dreisbach C., Alhusen J., Grossmann T. (2021). Gut microbiota composition is associated with newborn functional brain connectivity and behavioral temperament. Brain, Behav., Immun..

[bib25] Konvalinka I., Xygalatas D., Bulbulia J., Schjødt U., Jegindø E.-M., Wallot S., Orden G.V., Roepstorff A. (2011). Synchronized arousal between performers and related spectators in a fire-walking ritual. Proc. Natl. Acad. Sci..

[bib26] Lane R.D., McRae K., Reiman E.M., Chen K., Ahern G.L., Thayer J.F. (2009). Neural correlates of heart rate variability during emotion. NeuroImage.

[bib27] Maren S., Phan K.L., Liberzon I. (2013). The contextual brain: implications for fear conditioning, extinction and psychopathology. Nat. Rev. Neurosci..

[bib28] Markova G. (2018). The games infants play: social games during early mother–infant interactions and their relationship with oxytocin. Front. Psychol..

[bib29] Markova G., Nguyen T. (2022). Interpersonal synchrony is associated with infants’ reactions to subtle changes in caregiver‐infant interactions. *Social Development*, sode.

[bib30] Menon V. (2011). Large-scale brain networks and psychopathology: a unifying triple network model. Trends Cogn. Sci..

[bib31] Mesman J., van IJzendoorn M.H., Bakermans-Kranenburg M.J. (2009). The many faces of the Still-Face Paradigm: A review and meta-analysis. Dev. Rev..

[bib32] Mischel W., Shoda Y., Rodriguez M.L. (1989). Delay of gratification in children. Science.

[bib33] Molavi B., Dumont G.A. (2012). Wavelet-based motion artifact removal for functional near-infrared spectroscopy. Physiol. Meas..

[bib34] Morales S., Beekman C., Blandon A.Y., Stifter C.A., Buss K.A. (2015). Longitudinal associations between temperament and socioemotional outcomes in young children: The moderating role of RSA and gender. Dev. Psychobiol..

[bib35] Nguyen T., Schleihauf H., Kayhan E., Matthes D., Vrtička P., Hoehl S. (2020). The effects of interaction quality for neural synchrony during mother-child problem solving. Cortex.

[bib36] Nguyen T., Hoehl S., Vrtička P. (2021). A guide to parent-child fnirs hyperscanning data processing and analysis. Sensors.

[bib37] Nguyen T., Abney D.H., Salamander D., Bertenthal B.I., Hoehl S. (2021). Proximity and touch are associated with neural but not physiological synchrony in naturalistic mother-infant interactions. NeuroImage.

[bib38] Nigg J.T. (2006). Temperament and developmental psychopathology. J. Child Psychol. Psychiatry.

[bib39] Ochsner K.N., Gross J.J. (2007). In Handbook of emotion regulation.

[bib40] Perlman S.B., Pelphrey K.A. (2010). Regulatory brain development: balancing emotion and cognition. Soc. Neurosci..

[bib41] Perlman S.B., Luna B., Hein T.C., Huppert T.J. (2014). FNIRS evidence of prefrontal regulation of frustration in early childhood. NeuroImage.

[bib42] Piazza E., Hasenfratz L., Hasson U., Lew-Williams C. (2020). Infant and adult brains are coupled to the dynamics of natural communication. Psychol. Sci..

[bib43] Porges S.W. (1995). Orienting in a defensive world: mammalian modifications of our evolutionary heritage. A Polyvagal Theory. Psychophysiology.

[bib44] Porges S.W., Bohrer R.E. (1990). Principles of psychophysiology: Physical, social, and inferential elements.

[bib45] Porges S.W., Furman S.A. (2011). The early development of the autonomic nervous system provides a neural platform for social behavior: a polyvagal perspective. Infant Child Dev..

[bib46] Porter C.L. (2003). Coregulation in mother-infant dyads: links to infants’ cardiac vagal tone. Psychol. Rep..

[bib47] Porter C.L., Bryan Y.E., Hsu H.-C. (1995). Physiological markers in early infancy: stability of 1-to 6-month vagal tone. Infant Behav. Dev..

[bib48] Putnam S.P., Helbig A.L., Gartstein M.A., Rothbart M.K., Leerkes E. (2014). Development and assessment of short and very short forms of the infant behavior questionnaire–revised. J. Personal. Assess..

[bib49] Redcay E., Schilbach L. (2019). Using second-person neuroscience to elucidate the mechanisms of social interaction. Nat. Rev. Neurosci..

[bib50] Reindl V., Wass S., Leong V., Scharke W., Wistuba S., Wirth C.L., Konrad K., Gerloff C. (2022). Multimodal hyperscanning reveals that synchrony of body and mind are distinct in mother-child dyads. NeuroImage.

[bib51] Rothbart M.K., Sheese B.E., Rueda M.R., Posner M.I. (2011). Developing Mechanisms of Self-Regulation in Early Life. Emot. Rev..

[bib52] Sachis P.N., Armstrong D.L., Becker L.E., Bryan A.C. (1982). Myelination of the human vagus nerve from 24 weeks postconceptional age to adolescence. J. Neuropathol. Exp. Neurol..

[bib53] Sakaki M., Yoo H.J., Nga L., Lee T.-H., Thayer J.F., Mather M. (2016). Heart rate variability is associated with amygdala functional connectivity with MPFC across younger and older adults. NeuroImage.

[bib54] Sheskin D.J., Lovric M. (2011). *International Encyclopedia of St*at*i*stical *Science*.

[bib55] Shockley K., Butwill M., Zbilut J.P., Webber C.L. (2002).

[bib56] Sidor A., Fischer C., Cierpka M. (2017). The link between infant regulatory problems, temperament traits, maternal depressive symptoms and children’s psychopathological symptoms at age three: a longitudinal study in a German at-risk sample. Child Adolesc. Psychiatry Ment. Health.

[bib57] Tang A., Crawford H., Morales S., Degnan K.A., Pine D.S., Fox N.A. (2020). Infant behavioral inhibition predicts personality and social outcomes three decades later. Proc. Natl. Acad. Sci..

[bib58] Thayer J.F., Hansen A.L., Saus-Rose E., Johnsen B.H. (2009). Heart rate variability, prefrontal neural function, and cognitive performance: the neurovisceral integration perspective on self-regulation, adaptation, and health. Ann. Behav. Med.: A Publ. Soc. Behav. Med..

[bib59] Thomas E., Buss C., Rasmussen J.M., Entringer S., Ramirez J.S.B., Marr M., Rudolph M.D., Gilmore J.H., Styner M., Wadhwa P.D., Fair D.A., Graham A.M. (2019). Newborn amygdala connectivity and early emerging fear. Dev. Cogn. Neurosci..

[bib60] Tronick E.Z., Als H., Adamson L., Wise S., Brazelton T.B. (1978). The infant’s response to entrapment between contradictory messages in face-to-face interaction. J. Am. Acad. Child Psychiatry.

