# Supplementary material for: Coupling between prefrontal brain activity and respiratory sinus arrhythmia in infants and adults
Source: Dev Cogn Neurosci. 2022 Nov 30;58:101184. doi: 10.1016/j.dcn.2022.101184 (PMC9730144; doi:10.1016/j.dcn.2022.101184)
Supplement: Supplementary file 1 — Supplementary material [file mmc1.docx]

**Supplementary Information to manuscript:**

**“Coupling between Prefrontal Brain Activity and Respiratory Sinus Arrhythmia in Infants and Adults”**

Supplementary analyses

In further analyses, we compared recurrence rates between observed PFC(hbr)-RSA coupling and random coupling using the same models previously used on PFC(hbo)-RSA coupling. Model HbR1 tested observed against random coupling in infants. The model output showed no significant differences between observed and random coupling, *p* > .652. Model HbR 2 tested observed PFC-RSA coupling to random coupling adults. The model output revealed no significant differences, *p* = .050.

In addition, we applied the same models to PFC(hbr)-RSA coupling during the free play condition. While Model FP-HbR1 displayed a significant pairing effect, Χ^2^(1) = 5.944, *p* = .015, post-hoc analyses showed that observed coupling was lower than random coupling, *estimate* = -0.052, *SE* = 0.021, 95%CI = [-0.094 -0.010]. Model FP-HbR2 revealed no significant PFC-RSA coupling beyond random coupling, *p* = .258.

Supplementary Table

Table S1. Descriptive statistics of dependent variables (RSA, brain activity and max. recurrence)

| Variables | *M* | *SD* | *min* | *max* |
| --- | --- | --- | --- | --- |
| Baseline Condition | | | | |
| Infant | | | | |
| RSA | 4.12 | 0.78 | 2.65 | 5.83 |
| Brain activity (HbO) in IFG | 0.00 | 0.06 | -0.16 | 0.14 |
| Brain activity (HbO) in lPFC | 0.01 | 0.04 | -0.08 | 0.09 |
| Brain activity (HbO) in mPFC | 0.01 | 0.05 | -0.14 | 0.08 |
| Max. recurrence IFG-RSA | 0.03 | 0.01 | 0.02 | 0.06 |
| Max. recurrence lPFC-RSA | 0.03 | 0.01 | 0.02 | 0.05 |
| Max. recurrence mPFC-RSA | 0.03 | 0.01 | 0.02 | 0.05 |
|  |  |  |  |  |
| Brain activity (HbR) in IFG | 0.00 | 0.04 | -0.11 | 0.12 |
| Brain activity (HbR) in lPFC | 0.01 | 0.03 | -0.04 | 0.13 |
| Brain activity (HbR) in mPFC | 0.00 | 0.04 | -0.17 | 0.14 |
| Max. recurrence IFG-RSA | 0.03 | 0.01 | 0.02 | 0.05 |
| Max. recurrence lPFC-RSA | 0.03 | 0.01 | 0.02 | 0.07 |
| Max. recurrence mPFC-RSA | 0.03 | 0.01 | 0.02 | 0.07 |

| Variables | *M* | *SD* | *min* | *max* |
| --- | --- | --- | --- | --- |
| Mother | | | | |
| RSA | 5.61 | 1.20 | 2.64 | 8.13 |
| Brain activity (HbO) in IFG | 0.03 | 0.18 | -0.36 | 0.84 |
| Brain activity (HbO) in lPFC | 0.00 | 0.10 | -0.21 | 0.61 |
| Brain activity (HbO) in mPFC | 0.01 | 0.19 | -0.44 | 0.85 |
| Max. recurrence IFG-RSA | 0.03 | 0.01 | 0.01 | 0.06 |
| Max. recurrence lPFC-RSA | 0.03 | 0.01 | 0.02 | 0.04 |
| Max. recurrence mPFC-RSA | 0.03 | 0.01 | 0.02 | 0.05 |
|  |  |  |  |  |
| Brain activity (HbR) in IFG | -0.01 | 0.08 | -0.23 | 0.14 |
| Brain activity (HbR) in lPFC | 0.00 | 0.04 | -0.21 | 0.08 |
| Brain activity (HbR) in mPFC | 0.02 | 0.13 | -0.33 | 0.64 |
| Max. recurrence IFG-RSA | 0.03 | 0.01 | 0.02 | 0.05 |
| Max. recurrence lPFC-RSA | 0.03 | 0.01 | 0.02 | 0.04 |
| Max. recurrence mPFC-RSA | 0.03 | 0.01 | 0.01 | 0.05 |

| Variables | *M* | *SD* | *min* | *max* |
| --- | --- | --- | --- | --- |
| Free Play Condition | | | | |
| Infant | | | | |
| RSA | 4.37 | 1.14 | 2.49 | 8.50 |
| Brain activity (HbO) in IFG | -0.01 | 0.06 | -0.12 | 0.15 |
| Brain activity (HbO) in lPFC | 0.01 | 0.06 | -0.11 | 0.18 |
| Brain activity (HbO) in mPFC | 0.02 | 0.07 | -0.15 | 0.15 |
| Max. recurrence IFG-RSA | 0.05 | 0.01 | 0.03 | 0.08 |
| Max. recurrence lPFC-RSA | 0.05 | 0.01 | 0.03 | 0.09 |
| Max. recurrence mPFC-RSA | 0.05 | 0.01 | 0.03 | 0.07 |
|  |  |  |  |  |
| Brain activity (HbR) in IFG | 0.01 | 0.07 | -0.07 | 0.25 |
| Brain activity (HbR) in lPFC | 0.00 | 0.03 | -0.06 | 0.10 |
| Brain activity (HbR) in mPFC | -0.01 | 0.03 | -0.12 | 0.09 |
| Max. recurrence IFG-RSA | 0.05 | 0.01 | 0.03 | 0.08 |
| Max. recurrence lPFC-RSA | 0.05 | 0.01 | 0.03 | 0.09 |
| Max. recurrence mPFC-RSA | 0.05 | 0.01 | 0.02 | 0.08 |

| Variables | *M* | *SD* | *min* | *max* |
| --- | --- | --- | --- | --- |
| Mother | | | | |
| RSA | 5.74 | 1.10 | 3.03 | 8.55 |
| Brain activity (HbO) in IFG | 0.02 | 0.11 | -0.21 | 0.55 |
| Brain activity (HbO) in lPFC | 0.01 | 0.08 | -0.13 | 0.32 |
| Brain activity (HbO) in mPFC | -0.04 | 0.10 | -0.35 | 0.14 |
| Max. recurrence IFG-RSA | 0.05 | 0.01 | 0.02 | 0.07 |
| Max. recurrence lPFC-RSA | 0.05 | 0.01 | 0.03 | 0.10 |
| Max. recurrence mPFC-RSA | 0.05 | 0.01 | 0.03 | 0.07 |
|  |  |  |  |  |
| Brain activity (HbR) in IFG | -0.02 | 0.07 | -0.33 | 0.15 |
| Brain activity (HbR) in lPFC | 0.01 | 0.05 | -0.11 | 0.14 |
| Brain activity (HbR) in mPFC | 0.03 | 0.12 | -0.37 | 0.32 |
| Max. recurrence IFG-RSA | 0.05 | 0.01 | 0.02 | 0.09 |
| Max. recurrence lPFC-RSA | 0.05 | 0.01 | 0.03 | 0.06 |
| Max. recurrence mPFC-RSA | 0.05 | 0.01 | 0.02 | 0.07 |

Table S2. Descriptive statistics of fixed effect variables

| Variables | *M* | *SD* | *min* | *max* |
| --- | --- | --- | --- | --- |
| Infant | | | | |
| Age (months) | 4.8 | 0.5 | 4.03 | 6.00 |
| Infant temperament (negative affectivity) | 3.84 | 1.10 | 1.67 | 6.42 |
| Infant temperament (surgency) | 4.86 | 0.87 | 3.08 | 6.46 |
| Infant temperament (effortful control) | 5.30 | 0.67 | 3.75 | 6.67 |
| Infant negative affect during free play | 0.14 | 0.18 | 0.00 | 0.58 |
| Infant positive affect during free play | 0.25 | 0.21 | 0.00 | 0.81 |
|  |  |  |  |  |
| Caregiver | | | | |
| Age | 33.97 | 4.40 | 24 | 44 |

Table S3. Correlation matrix of fixed effect variables

| Variables | *1* | *2* | *3* | *4* | *5* | *6* | *7* | *8* | *9* |
| --- | --- | --- | --- | --- | --- | --- | --- | --- | --- |
| Infant age | 1 | - |  |  |  |  |  |  |  |
| Caregiver age | - | 1 |  |  |  |  |  |  |  |
| Infant basal RSA | 0.34** | 0.12 | 1 |  |  |  |  |  |  |
| Caregiver basal RSA | -0.02 | -0.00 | 0.05 | 1 |  |  |  |  |  |
| Infant temperament (negative affectivity) | 0.08 | 0.19 | 0.05 | -0.08 | 1 |  |  |  |  |
| Infant temperament (surgency) | 0.21 | 0.02 | 0.08 | -0.04 | 0.35* | 1 |  |  |  |
| Infant temperament (effortful control) | -0.07 | 0.04 | 0.20 | -0.29* | -0.05 | -0.33* | 1 |  |  |
| Infant positive affect during free play | 0.01 | -0.21 | -0.09 | 0.03 | -0.04 | 0.29* | -0.09 | 1 |  |
| Infant negative affect during free play | 0.02 | 0.07 | -0.02 | 0.12 | -0.11 | -0.27 | 0.05 | **-0.51***** | 1 |

Notes. *** < 0.001, ** < 0.01, * < 0.05. Bold marked correlation coefficient remains significant after Bonferroni correction.

Table S4. Model outputs (Models 1-5)

|  | *Estimates* | *SE* | *CI Lower* | *CI Upper* | *X²* | *df* | *p* |
| --- | --- | --- | --- | --- | --- | --- | --- |
| Infant Model 1: *MaxRec ~ region + type + region : type + (1 \| RSA.ID)* | | | | | | | |
| (Intercept) | -3.503 | 0.027 | -3.556 | -3.450 |  |  |  |
| region |  |  |  |  | 1.646 | 2 | .439 |
| region (lPFC) | 0.041 | 0.034 | -0.026 | 0.109 |  |  |  |
| region (mPFC) | 0.033 | 0.034 | -0.034 | 0.099 |  |  |  |
| type |  |  |  |  | 1.596 | 1 | .206 |
| type (surrogate) | 0.037 | 0.034 | -0.029 | 0.103 |  |  |  |
| region : type |  |  |  |  | 0.389 | 2 | .823 |
| region (lPFC) : type (surrogate) | -0.027 | 0.045 | -0.117 | 0.061 |  |  |  |
| region (mPFC) : type (surrogate) | -0.012 | 0.045 | -0.100 | 0.077 |  |  |  |

|  | *Estimates* | *SE* | *CI Lower* | *CI Upper* | *X²* | *df* | *p* |
| --- | --- | --- | --- | --- | --- | --- | --- |
| Caregiver Model 1: *MaxRec ~ region + type + region : type + (1 \| RSA.ID)* | | | | | | | |
| (Intercept) | -3.505 | 0.024 | -3.553 | -3.458 |  |  |  |
| region |  |  |  |  | 0.395 | 2 | .820 |
| region (lPFC) | -0.034 | 0.032 | -0.097 | 0.028 |  |  |  |
| region (mPFC) | -0.011 | 0.032 | -0.074 | 0.051 |  |  |  |
| type |  |  |  |  | 2.550 | 1 | .110 |
| type (surrogate) | 0.013 | 0.030 | -0.045 | 0.073 |  |  |  |
| region : type |  |  |  |  | 0.919 | 2 | .635 |
| region (lPFC) : type (surrogate) | 0.038 | 0.042 | -0.045 | 0.121 |  |  |  |
| region (mPFC) : type (surrogate) | 0.006 | 0.043 | -0.078 | 0.089 |  |  |  |

|  | *Estimates* | *SE* | *CI Lower* | *CI Upper* | *X²* | *df* | *p* |
| --- | --- | --- | --- | --- | --- | --- | --- |
| Infant Model 2: *MaxRec ~region + negaff + surgency + effortfulcontrol +* infant positive affect *+ infant negative affect (dur) + (1 \| RSA.ID)* | | | | | | | |
| (Intercept) | -3.253 | 0.250 | -3.842 | -2.864 |  |  |  |
| region |  |  |  |  | 5.450 | 2 | .066 |
| region (lPFC) | 0.125 | 0.054 | 0.020 | 0.230 |  |  |  |
| region (mPFC) | 0.082 | 0.054 | -0.023 | 0.187 |  |  |  |
| negaff | -0.022 | 0.025 | -0.072 | 0.028 | 0.733 | 1 | .392 |
| surgency | -0.035 | 0.028 | -0.090 | 0.019 | 1.552 | 1 | .213 |
| effortfulcontrol | 0.016 | 0.033 | -0.049 | 0.082 | 0.245 | 1 | .620 |
| infant positive affect (dur) | -0.062 | 0.118 | -0.294 | 0.170 | 0.273 | 1 | .601 |
| infant negative affect (dur) | -0.079 | 0.152 | -0.376 | 0.218 | 0.272 | 1 | .602 |

|  | *Estimates* | *SE* | *CI Lower* | *CI Upper* | *X²* | *df* | *p* |
| --- | --- | --- | --- | --- | --- | --- | --- |
| Infant Model 3: *MaxRec ~ region + basal rsa + age + (1 \| RSA.ID)* | | | | | | | |
| (Intercept) | -3.467 | 0.183 | -3.827 | -3.107 |  |  |  |
|  | *Estimates* | *SE* | *CI Lower* | *CI Upper* | *X²* | *df* | *p* |
| region |  |  |  |  | 0.869 | 2 | .647 |
| region (lPFC) | 0.045 | 0.050 | -0.052 | 0.143 |  |  |  |
| region (mPFC) | 0.035 | 0.049 | -0.062 | 0.132 |  |  |  |
| basal rsa | -0.048 | 0.025 | -0.096 | 0.000 | 3.791 | 1 | .051 |
| age | 0.000 | 0.001 | -0.002 | 0.003 | 0.489 | 1 | .484 |

|  | *Estimates* | *SE* | *CI Lower* | *CI Upper* | *X²* | *df* | *p* |
| --- | --- | --- | --- | --- | --- | --- | --- |
| Adult Model 3: *MaxRec ~ region + basal rsa + (1 \| RSA.ID)* | | | | | | | |
| (Intercept) | -3.646 | 0.104 | -3.852 | -3.442 |  |  |  |
| region |  |  |  |  | 0.168 | 2 | .919 |
| region (lPFC) | -0.018 | 0.046 | -0.109 | 0.072 |  |  |  |
| region (mPFC) | -0.006 | 0.466 | -0.097 | 0.086 |  |  |  |
| basal rsa | 0.024 | 0.017 | -0.010 | 0.058 | 1.870 | 1 | .171 |

Supplementary Figure

*
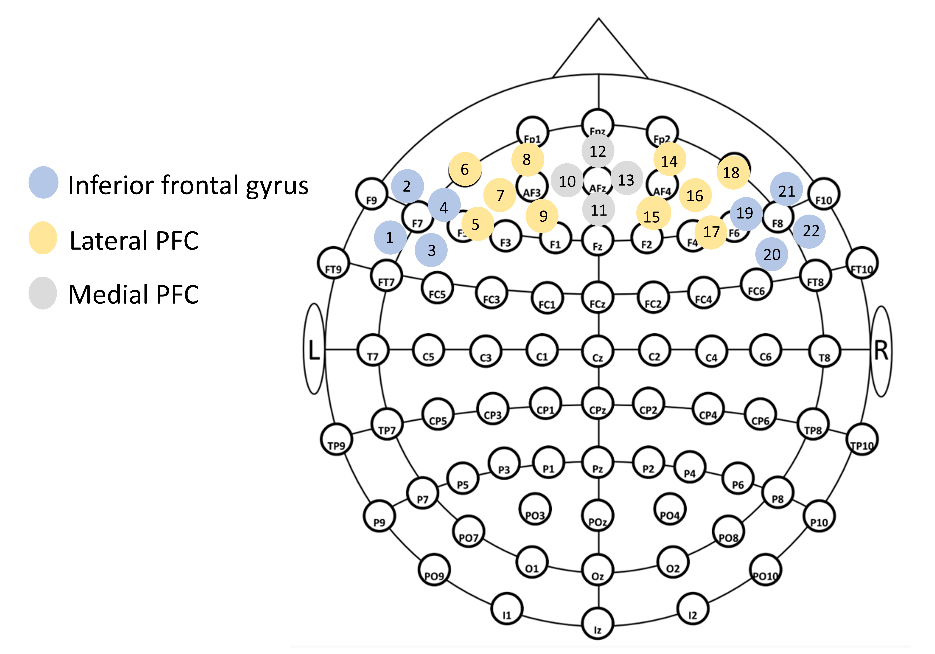
*

*Figure S1.* The channel configuration of the mother and infant cap comprises the following brain areas: Inferior frontal gyrus (1-4; 19-22), lateral prefrontal cortex (PFC; 5-9; 14-18), and medial prefrontal cortex (10-13).


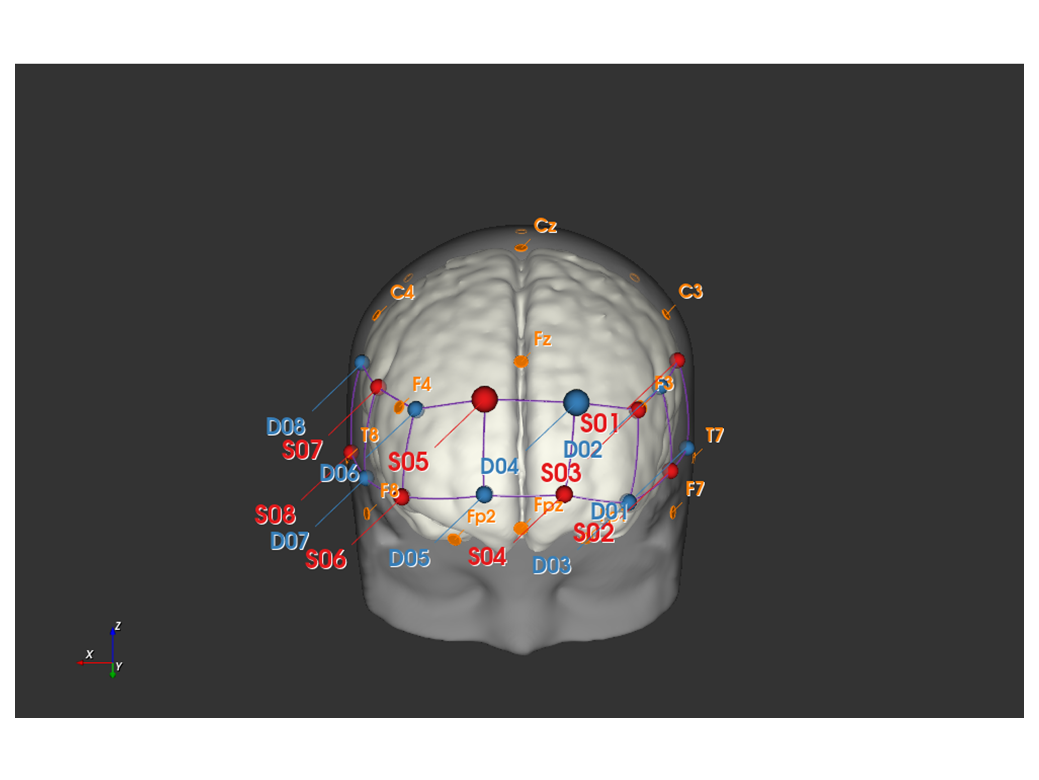

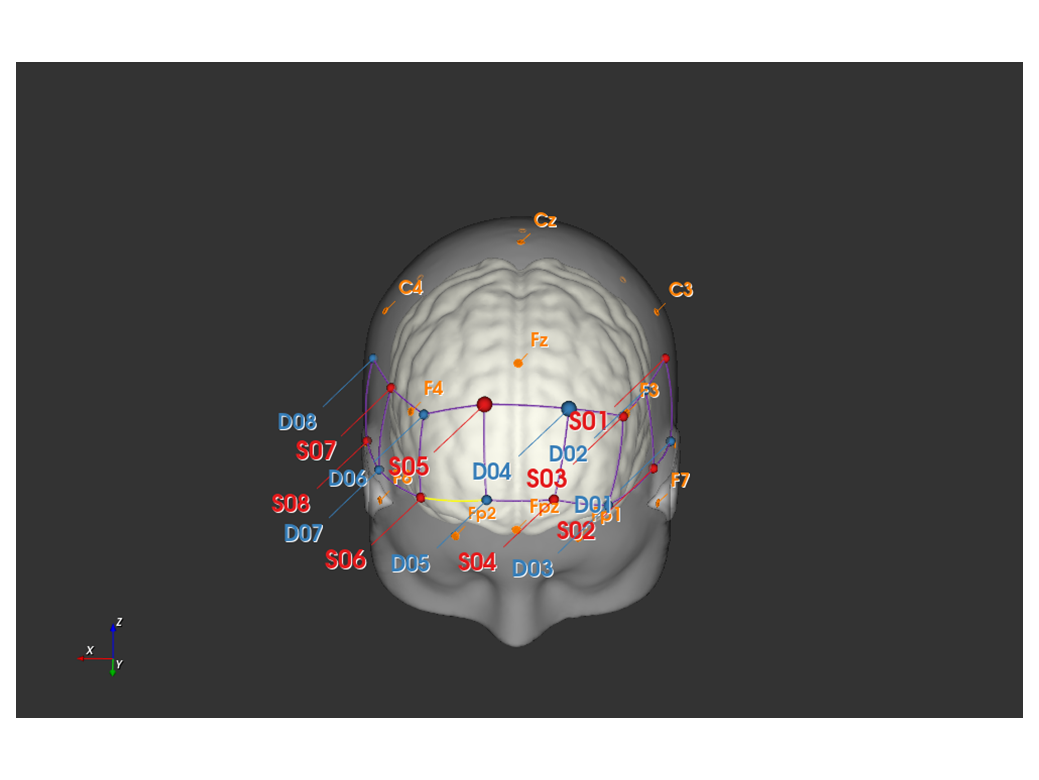


*Figure S2.* Optode positions are projected onto a 2-5-month-old infant head model (Infant Atlases 0-4.5 years; top) and an adult head model (ICBM 152 Nonlinear atlases version 2009; bottom). EEG 10/20 reference labels, which we used to place optode positions onto the caps, are marked in orange. Sources are in red, and detectors are in blue. Projections were implemented using NIRSite 2021.4.
